# Supplementary material for: Development and External Validation of a Radiomics Model Derived from Preoperative Gadoxetic Acid-Enhanced MRI for Predicting Histopathologic Grade of Hepatocellular Carcinoma
Source: Diagnostics (Basel). 2023 Jan 23;13(3):413. doi: 10.3390/diagnostics13030413 (PMC9914153; doi:10.3390/diagnostics13030413)
Supplement: Supplementary file 1 [file diagnostics-13-00413-s001.zip › diagnostics-2136689-si.pdf]

Supplementary materials

Development and External Validation of a Radiomics Model Derived from Preoperative Gadoxetic Acid-Enhanced MRI for Predicting Histopathologic Grade of Hepatocellular Carcinoma

Table S1. Gadoxetic acid-enhanced MRI scanning protocol at two participating cneters

Table S1A. Gadoxetic acid-enhanced MRI scanning parameters in the development cohort

Phase	Repitition time (ms)	Echo time (ms)	Field of view (mm×mm)	Flip angle (degree)	Slice thickness (mm)
Arterial phase	3.7	1.31	330×241	10	2.5
Portal vein phase	3.2	1.13	240×330	10	2.5
Delayed phase	3.2	1.13	240×330	10	2.5
Hepatobiliary phase	3.2	1.13	240×330	10	3.5

Table S1B. Gadoxetic acid-enhanced MRI scanning parameters in the test cohort

Phases	Repetition time (ms)	Echo time (ms)	Field of view (mm×mm)	Flip angle (degree)	Slice thickness (mm)
Arterial phase	3.42	1.25	400×400	13	2.5
Portal vein phase	3.42	1.25	400×400	13	2.5
Delayed phase	3.42	1.25	400×400	13	2.5
Hepatobiliary phase	3.42	1.25	400×400	30	2.5

Table S2. Radiomics features included in the three radiomics models

Logistic regression model (22):

Radiomics features
original_firstorder_Energy
original_firstorder_Mean
original_glszm_SmallAreaHighGrayLevelEmphasis
original_gldm_GrayLevelNonUniformity
original_glcm_JointEnergy
wavelet-HLL_firstorder_Kurtosis
wavelet-HLL_glrlm_GrayLevelVariance
wavelet-HLH_glcm_ClusterTendency
wavelet-HLH_glcm_Correlation
wavelet-HLH_glcm_DifferenceVariance
wavelet-HLH_glcm_MaximumProbability
wavelet-HLH_glcm_SumEntropy
wavelet-HHL_firstorder_Mean
wavelet-HHL_glcm_Correlation
wavelet-HHL_glcm_JointEnergy
wavelet-HHL_glrlm_GrayLevelVariance
wavelet-HHH_glcm_Correlation
wavelet-HHH_glcm_MaximumProbability
wavelet-LLL_firstorder_Maximum
wavelet-LLL_firstorder_Minimum
wavelet-LLL_gldm_HighGrayLevelEmphasis
wavelet-LLL_glcm_DifferenceVariance

Support vector machine (22):

Radiomics features
original_firstorder_Energy
original_firstorder_Mean
original_glszm_SmallAreaHighGrayLevelEmphasis
original_gldm_GrayLevelNonUniformity
original_glcm_JointEnergy
wavelet-HLL_firstorder_Kurtosis
wavelet-HLL_glrlm_GrayLevelVariance
wavelet-HLH_glcm_ClusterTendency
wavelet-HLH_glcm_Correlation
wavelet-HLH_glcm_DifferenceVariance
wavelet-HLH_glcm_MaximumProbability
wavelet-HLH_glcm_SumEntropy

wavelet-HHL_firstorder_Mean
wavelet-HHL_glcm_Correlation
wavelet-HHL_glcm_JointEnergy
wavelet-HHL_glrlm_GrayLevelVariance
wavelet-HHH_glcm_Correlation
wavelet-HHH_glcm_MaximumProbability
wavelet-LLL_firstorder_Maximum
wavelet-LLL_firstorder_Minimum
wavelet-LLL_gldm_HighGrayLevelEmphasis
wavelet-LLL_glcm_DifferenceVariance

Adaboost model (10):

**Radiomics features**
original_firstorder_Energy
original_glcm_JointEnergy
wavelet-HLH_glcm_ClusterTendency
wavelet-HLH_glcm_DifferenceVariance
wavelet-HLH_glcm_SumEntropy
wavelet-HHL_firstorder_Mean
wavelet-HHL_glcm_Correlation
wavelet-HHL_glcm_JointEnergy
wavelet-HHH_glcm_MaximumProbability
wavelet-LLL_gldm_HighGrayLevelEmphasis

Table S3. Parameters in the development of the model with support vector machine, and Adaboost.

Key parameters in support vector machine classifier: kernel = “rbf”, degree = 3, gamma = “scale”, tol = 1e-3, cache_size = 200. Others used as default.
Key parameters in the Adaboost classifier: n_estimators = 50, learning_rate = 1.0, algorithm = “SAMME.R”, base_estimators = None. Others used as default.

Table S4. Formula of the logistic regression radiomics model

Y = 0.261
-0.465*original_firstorder_Energy+
0.662*original_firstorder_Mean+
0.081*original_glszm_SmallAreaHighGrayLevelEmphasis+
0.069*original_gldm_GrayLevelNonUniformity
-0.408*original_glcm_JointEnergy+
0.224*wavelet-HLL_firstorder_Kurtosis+
0.567*wavelet-HLL_glrlm_GrayLevelVariance
-0.095*wavelet-HLH_glcm_ClusterTendency+
0.089*wavelet-HLH_glcm_Correlation
-0.212*wavelet-HLH_glcm_DifferenceVariance
-0.274*wavelet-HLH_glcm_MaximumProbability
-0.151*wavelet-HLH_glcm_SumEntropy
-0.556*wavelet-HHL_firstorder_Mean
-0.217*wavelet-HHL_glcm_Correlation
-0.278*wavelet-HHL_glcm_JointEnergy
-0.128*wavelet-HHL_glrlm_GrayLevelVariance+
0.151*wavelet-HHH_glcm_Correlation+
0.693*wavelet-HHH_glcm_MaximumProbability+
0.116*wavelet-LLL_firstorder_Maximum+
0.152*wavelet-LLL_firstorder_Minimum
-0.19*wavelet-LLL_gldm_HighGrayLevelEmphasis
-0.178*wavelet-LLL_glcm_DifferenceVariance
